# Supplementary material for: Causal Relationship between Aging and Anorexia Nervosa: A White-Matter-Microstructure-Mediated Mendelian Randomization Analysis
Source: Biomedicines. 2024 Aug 16;12(8):1874. doi: 10.3390/biomedicines12081874 (PMC11351342; doi:10.3390/biomedicines12081874)
Supplement: Supplementary file 1 [file biomedicines-12-01874-s001.zip › Supplementary Figure/Figure S8/Leaveoneout plot of MR analysis for TL to ubm-b-1719.pdf]

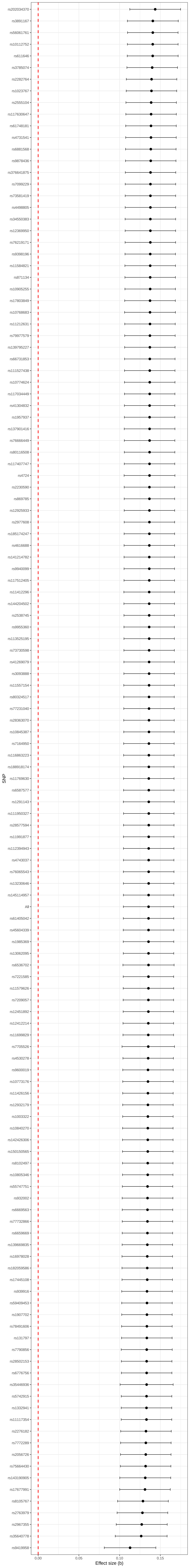
rs202034370
rs3891167
rs56061761
rs10112752
rs611646
rs3785074
rs2282764
rs1023767
rs2555104
rs117630647
rs61748181
rs4731541
rs6881568
rs9878436
rs376641875
rs7099229
rs73581419
rs4498805
rs34550383
rs12369950
rs76219171
rs9398196
rs11584821
rs871134
rs10905255
rs17803849
rs10768683
rs11212631
rs79977579
rs139795227
rs66731853
rs111527438
rs10774624
rs117034449
rs41304832
rs1957937
rs137901416
rs76666449
rs80116508
rs117407747
rs4724
rs2230590
rs869785
rs12925933
rs2977608
rs185174247
rs4616688
rs141214782
rs9940099
rs117512405
rs11412296
rs144204502
rs2538745
rs9955360
rs113525195
rs73730598
rs41269079
rs3093888
rs11557154
rs80324517
rs77231040
rs28363070
rs10845387
rs7164950
rs116863223
rs188918174
rs11769630
rs6587577
rs1291143
rs111950327
rs28577594
rs11991877
rs112394943
rs4743037
rs76065543
rs13230646
rs145114957
All
rs61405042
rs45604339
rs1985369
rs13062095
rs6536702
rs7221585
rs11579626
rs7209057
rs12451892
rs12412214
rs11699829
rs7705526
rs4530278
rs9600019
rs10773176
rs11426156
rs12932179
rs1003322
rs10840270
rs142426306
rs150150565
rs8102497
rs10805346
rs55747751
rs932002
rs6669563
rs77732866
rs6659669
rs139669835
rs16978028
rs182059586
rs17445108
rs939916
rs59409453
rs1907702
rs78491606
rs131797
rs7790856
rs28502153
rs6776756
rs35446936
rs5742915
rs1332941
rs11117354
rs2276182
rs7772289
rs2056726
rs75664430
rs143190905
rs17677991
rs8105767
rs2763979
rs2967355
rs35640778
rs9419958
SNP
0.00
0.05
0.10
0.15
Effect size (b)
